# Opana ER (Oxymorphone)–Induced Thrombotic Microangiopathy: An Atypical Presentation in a Patient With Hepatitis C

**DOI:** 10.1177/2324709618756423

**Published:** 2018-01-31

**Authors:** Hassan Mehmood, Muzammil Khan, Asghar Marwat, Medha Joshi, Varun Malhotra

**Affiliations:** 1 Temple University/Conemaugh Memorial Medical Center, Johnstown, PA, USA

**Keywords:** Opana ER, thrombotic microangiopathy, ADAMTS-13, kidney biopsy, hepatitis C

## Abstract

Oxymorphone is a semisynthetic extended release opiate used to treat moderate to severe chronic pain. The Food and Drug Administration approved the oral form of oxymorphone available as Opana and Opana ER (extended release) since 2006. The Food and Drug Administration and the Centers for Disease Control and Prevention issued warning against intravenous use of Opana ER. We are presenting a case report of a 37-year-old female with a history of active intravenous drug abuse who presented to our hospital with acute kidney injury. Urinalysis showed red blood cell sediments, many dysmorphic red blood cell casts along with nephrotic range proteinuria of 12 g/deal per day. Kidney biopsy showed microscopic thrombotic microangiopathy (TMA) involving glomeruli and vessels. Further workup was undertaken for TMA, and apart from mildly elevated lactate dehydrogenase of 380 (normal <243), active hepatitis C, and slightly low ADAMTS-13 (55%), there was no other laboratory evidence of TMA. On literature search, we found that intravenous injection of chronic Opana ER has been reported to cause TMA resulting in chronic kidney disease. Our patient also admitted to use of intravenous Opana ER abuse for the past 5 years. She had a normal platelet count and an absence of schistocytes, which makes it an atypical presentation of TMA resulting in chronic kidney disease in an opiate user. We strongly urge physicians to avoid prescribing opiates for chronic pain, especially Opana ER, which if injected intravenously for recreational purposes can lead to serious side effects like TMA. Treatment is mainly supportive and avoidance of drug in future.

## Introduction

Oxymorphone is a semisynthetic extended release opiate used to treat moderate to severe chronic pain.^1^ The Food and Drug Administration approved the oral form of oxymorphone available as Opana and Opana ER (extended release) since 2006. Despite the warning issued by the Food and Drug Administration and the Centers for Disease Control and Prevention, Opana ER continues to be used by recreational users in intravenous (IV) form leading to severe adverse effects like thrombotic microangiopathy (TMA). In 2012, even though crush-resistant formulation was introduced by pharmaceutical companies, IV drug users continue to inject the drug in melted form.^2,3^

## Case Description

**Figure 1.**
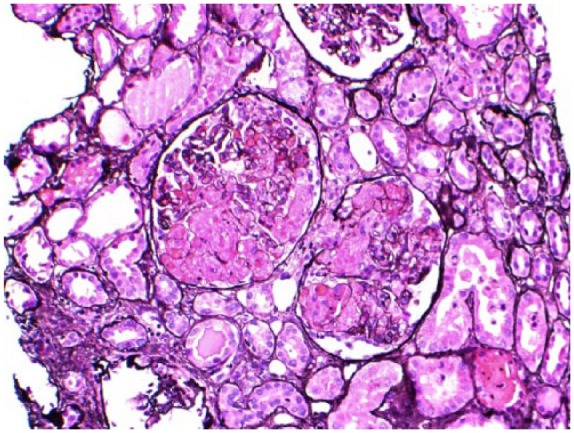
Thrombotic microangiopathy involving vessels and glomeruli.

**Figure 2.**
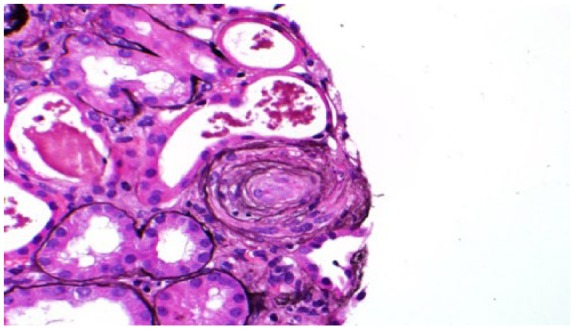
Thrombotic microangiopathy involving vessels and glomeruli.

We in this article, present an interesting case of a 37-year-old female with a history of active IV drug abuse and asthma who presented to our hospital for shortness of breath of 3 weeks duration. Clinical examination revealed blood pressure of 150/78 mm Hg, pulse of 108 per minute, and temperature of 95°F, and the rest of the examination was unremarkable. Echocardiography findings were consistent with acute diastolic congestive heart failure that was managed with IV diuretics. Her laboratory results suggested renal failure (creatinine 2.2 mg/dL) with baseline creatinine of 1.8 mg/dL documented 6 months before this presentation. Urinalysis showed red blood cell sediments, many dysmorphic red blood cell casts along with nephrotic range proteinuria of 12 g/dL per day. She also had mild proteinuria of 100 mg/dL 6 months ago. Kidney biopsy was obtained that suggested microscopic TMA involving the glomeruli and vessels (Figures 1 and 2). Eighty-four glomeruli were reviewed on light microscope, 28 of which were globally sclerotic and some had mesangiolysis. Most of the glomeruli showed ischemic-type wrinkling of glomerular basement membrane. There was erythrocytes congestion and fragmentation along with focal fibrin thrombi. There was mild to moderate tubular atrophy and interstitial fibrosis involving approximately 50% of the cortex samples. Eight glomeruli were sampled for immunofluorescence and 3 of them were globally sclerotic. There was 1+ granular global mesangial, glomerular capillary wall, and arteriolar staining for immunoglobulin (Ig) M. There was also 1+ granular mesangial staining for C3 with negative IgA, negative albumin, trace IgG, trace C1q, and trace lambda. Several arterioles also showed 3+ staining for fibrinogen on immunofluorescence. Electron microscopy did not show globally sclerosis among 4 survey blocks. Some glomeruli showed inframembranous hyalinosis. Glomerular basement membranes exhibit segmental ischemic-type wrinkling. Some glomerular loops showed mild widening of the subendothelial zone by electron lucent material. No endothelial tubuloreticular inclusions were seen. Few mesangial areas exhibited mesangial electron dense deposits. Subendothelial electron dense deposits were seen along with double contour basement membrane. Further workup was undertaken for TMA, and apart from mildly elevated lactate dehydrogenase of 380 (normal <243) and slightly low ADAMTS-13 (55%), there was no other laboratory evidence of TMA with normal platelet count, normal haptoglobin, mildly high reticulocyte count, and no schistocytes in the peripheral smear. Scleroderma antibodies, antiphospholipid antibodies, anti-GBM antibodies, and C3 and C4 complement antineuclear antibodies were negative. Hepatitis B and HIV was negative and hepatitis C antibody was positive, but cryoglobulin was negative. On literature search, we found that IV injection of chronic Opana ER has been reported to cause TMA resulting in chronic kidney disease (CKD). Our patient admitted with a history of ongoing IV Opana ER use for the past 5 years; therefore, TMA was considered drug induced. The patient was only using diuretics and denied abusing any other drug. She had a normal platelet count and an absence of schistocytes, which makes it an atypical presentation of TMA without classic findings resulting in CKD secondary to chronic opiate user for 5 years. As there was no evidence of thrombotic thrombocytopenic purpura (TTP), plasma pheresis was not done and eventually ADAMTS-13 was not low enough to suggest TTP for that level should be less than 10%. The patient was offered opiate rehabilitation counseling; however, she left against medical advice.

## Discussion

Use of several medications like quinine, clopidogrel, interferon, gemcitabine, and vascular endothelial growth factors have been reported as a cause of TMA.^4^ Mechanism of injury for most these drugs is unknown. Some studies have shown TMA secondary to vascular endothelial growth factor involves renal products.^5,6^ Quinine may involve an immune process leading to endothelial cell injury.^7^

The cause of TMA due to Opana ER is not clear yet. It could be secondary to immune-mediated response or chronic dose-mediated effect.^4^ The Food and Drug Administration approved the ER form of Opana in 2006. The reformulated form of Opana ER has a polyethylene oxide and polyethylene glycol.^8^ Polyethylene oxide has been reported as a cause of dose-dependent hemolytic anemia and thrombocytopenia in rats following administration of intravenous and intraperitoneal injections.^9^ One study showed polyethylene oxide caused intravenous hemolysis and renal failure in guinea pigs.^10^

Underlying viral infection like hepatitis C could be an important factor in triggering injury causing TMAs. These viruses can affect endothelial dysfunction, leading to TMA.^11^ Our patient also had an active hepatitis C with a high viral load. The Centers for Disease Control and Prevention released a report of TTP-like illness secondary to Opana ER in Tennessee in 2013. Among those 15 patients, 12 had either chronic hepatitis C or had positive anti–hepatitis C virus antibodies.^12^ ADAMTS-13 and anticardiolipin antibodies are associated with development of hepatitis C virus–related TMA.^10^

The clinical presentation of drug-induced TMA due to Opana ER is similar to TTP or hemolytic-uremic syndrome. Microangiopathic hemolytic anemia, thrombocytopenia, fever, renal insufficiency, neurological symptoms, and TMA are the common findings. There is no specific test for diagnosis, and high degree of suspicion is the key in early diagnosis. ADAMTS-13 activity is mild to moderately decreased and can help in early diagnosis.^13-15^ ADAMTS-13 is less than 10% in TTP.^16^ Two fatalities were reported in 2009^17^ and focus was placed to improve screening test. Combination of DRI oxy assay and CEDIA Opi test can detect oxymorphone in urine.^18^

Supportive management along with cessation of drug is the recommended therapy and role of plasma exchange is yet to be determined. Previously reported cases were successfully treated with supportive management.^19^ Our patient was started on the aforementioned recommended therapy, but she left prematurely against medical advice.

## Conclusion

The opioid epidemic is a growing public health concern. Despite the efforts to curb opiate prescriptions, narcotic abuse has remained high in certain parts of the country. We strongly urge physicians to avoid prescribing opiates for chronic pain, especially Opana ER, which if injected intravenously for recreational purposes can lead to serious side effects like TMA. The diagnosis should be kept in mind in an injection drug user who presents with symptoms like TTP, hemolytic-uremic syndrome, and subacute or chronic kidney disease of unclear etiology. Early diagnosis is the key as Opana-induced TMA has a high mortality and morbidity along with serious hepatorenal pathology. We recommend health care professional to take a detailed history with focused questions on recent IV Opana ER abuse. In a nutshell, treatment is mainly supportive along with the treatment of underlying infection and avoidance of drug in the future that could be challenging in intravenous drug abusers like ours.
